# The impact of summer drought on peat soil microbiome structure and function-A multi-proxy-comparison

**DOI:** 10.1038/s43705-022-00164-x

**Published:** 2022-08-25

**Authors:** Haitao Wang, Mareike Meister, Corinna Jensen, Andreas W. Kuss, Tim Urich

**Affiliations:** 1 Institute of Microbiology, University of Greifswald, Greifswald, Germany; 2 Diabetes Competence Centre Karlsburg (KDK), Leibniz Institute for Plasma Science and Technology (INP), Karlsburg, Germany; 3 Human Molecular Genetics Group, Department of Functional Genomics, University Medicine Greifswald, Greifswald, Germany

**Keywords:** Microbiome, Microbial ecology

## Abstract

Different proxies for changes in structure and/or function of microbiomes have been developed, allowing assessing microbiome dynamics at multiple levels. However, the lack and differences in understanding the microbiome dynamics are due to the differences in the choice of proxies in different studies and the limitations of proxies themselves. Here, using both amplicon and metatranscriptomic sequencings, we compared four different proxies (16/18S rRNA genes, 16/18S rRNA transcripts, mRNA taxonomy and mRNA function) to reveal the impact of a severe summer drought in 2018 on prokaryotic and eukaryotic microbiome structures and functions in two rewetted fen peatlands in northern Germany. We found that both prokaryotic and eukaryotic microbiome compositions were significantly different between dry and wet months. Interestingly, mRNA proxies showed stronger and more significant impacts of drought for prokaryotes, while 18S rRNA transcript and mRNA taxonomy showed stronger drought impacts for eukaryotes. Accordingly, by comparing the accuracy of microbiome changes in predicting dry and wet months under different proxies, we found that mRNA proxies performed better for prokaryotes, while 18S rRNA transcript and mRNA taxonomy performed better for eukaryotes. In both cases, rRNA gene proxies showed much lower to the lowest accuracy, suggesting the drawback of DNA based approaches. To our knowledge, this is the first study comparing all these proxies to reveal the dynamics of both prokaryotic and eukaryotic microbiomes in soils. This study shows that microbiomes are sensitive to (extreme) weather changes in rewetted fens, and the associated microbial changes might contribute to ecological consequences.

Soil microbiomes are complex assemblages, consisting of both prokaryotic (bacteria and archaea) as well as eukaryotic (fungi, protists and metazoan) organisms [1] that interact in a multitude of ways critical for ecosystem functioning [2]. Correctly probing the dynamic microbiome response to abiotic and biotic factors is crucial for our understanding in times of global change. How to measure microbiome dynamics? Different proxies for changes in structure and/or function of microbiomes have been developed and applied. The amplicon sequencings of 16S and 18S rRNA genes are widely used to access total prokaryotic and eukaryotic communities, respectively. Next to the bias introduced by primers and amplification, the persistence of relic DNA from dead organisms can obscure microbial responses to environmental changes [3]. To mitigate this bias, rRNA transcripts have been employed as the proxy to target active organisms or living biomass. However, a new concern arises given that soils likely contain a large dormant fraction of organisms, which can contribute considerable amounts of rRNA [4]. Metatranscriptomics allowing the simultaneous assessment of rRNA and mRNA can alleviate the above-mentioned drawbacks, as mRNA reflecting the gene expression is metabolically incidental, providing a highly sensitive bioassay for environmental changes relevant to microbes [5]. This “double-RNA” approach also provides both taxonomic and functional insights in microbiome compositions [6]. While mRNA has been largely employed to interpret microbial functions, the taxonomy assigned with mRNA, which could be a good indicator of metabolically active microorganisms, is despised. To date, a comparison of all these proxies for assessing microbiome dynamics is still lacking.

Understanding the temporal dynamics of microbiomes in the course of drought in wetland ecosystems is important as such extreme weather now occurs more often due to climate change. However, there are still the lack and debates in understanding the temporal dynamics of microbiomes due to the differences in the choice of proxies in different studies and the limitations of proxies themselves. Rewetted fen peatlands are locally novel ecosystems [7], and microbiomes in these ecosystems are still under-characterized. A previous study showed the seasonal dynamics mainly in eukaryotic microbiomes in three rewetted fens [8], but it remains unclear if this pattern persists in the year with extreme weather events and if the chosen proxy matters in revealing microbiome dynamics.

Here, we delineated the impact of summer drought on the microbiome structure and function in two rewetted fen peatlands in northern Germany, namely coastal fen and percolation fen [9], with the comparison between four different microbiome proxies: (I) 16/18S rRNA genes, (II) 16/18S rRNA transcripts, (III) mRNA taxonomy, (IV) mRNA function, based on amplicon sequencing and metatranscriptomic sequencing. In 2018, a severe heatwave and drought hit Europe in the summer, with significantly higher temperature and less precipitation (from April to September) compared to previous years [10–12]. Consequently, this summer drought resulted in a significant decline in water level (down to around −80 and −20 cm) and an increase in redox potential (up to around 120 and 100 mV) in coastal and percolation fen, respectively (Fig. S1). Soil samples were taken in two-month intervals from April 2018 until February 2019 (Fig. 1a), covering the periods before, during and after the drought happened (Fig. S1). Both prokaryotic and eukaryotic microbiomes (Fig. 1b) were investigated with these proxies. The detailed methods are described in the Supplementary Information.

**Fig. 1:**
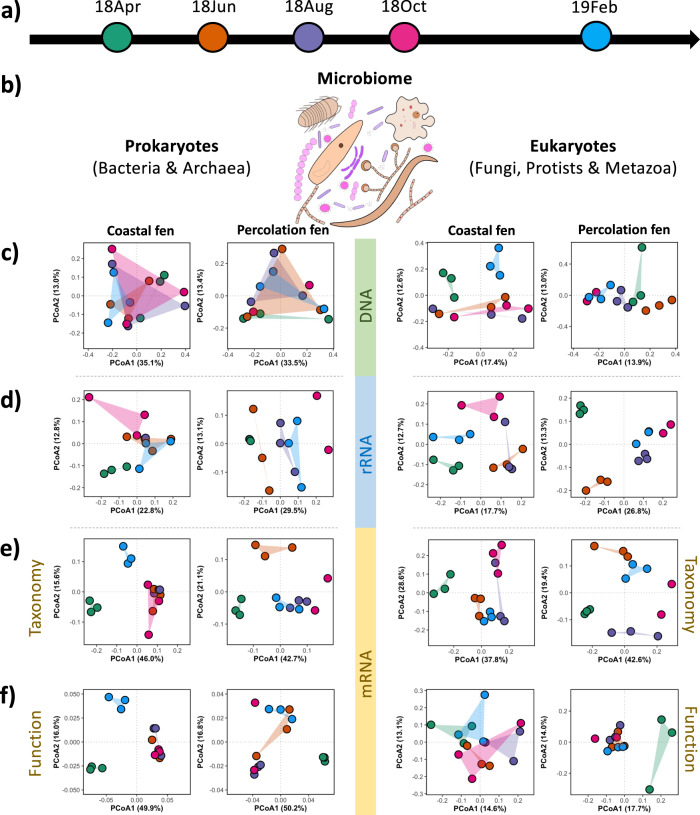
Changes in microbiome structures and function compositions. **a** Sampling timeline. **b** Microbiome composition. **c**–**f** PCoA showing changes in prokaryotic and eukaryotic microbiome structures and function compositions over the studying months based on 16/18S rRNA genes (**c**), 16/18S rRNA transcripts (**d**), mRNA taxonomy (**e**), mRNA function (**f**). The color codes in (**c**–**f**) correspond to color codes in (**a**).

To assess the drought impact, 18-Apr and 19-Feb were considered as wet months (water level ≥sampling depth, Fig. S1) while 18-Jun, 18-Aug and 18-Oct were considered as dry months (water level < sampling depth, Fig. S1). The principal coordinate analysis showed that, on DNA level, the difference in microbiome composition between dry and wet months was only significant in eukaryotes in the coastal fen (Fig. 1c), as characterized by permutational multivariate analysis of variance (Table S1). Surprisingly, mRNA proxies suggested strong and significant impacts of drought on prokaryotic compositions in contrast to rRNA proxies (Fig. 1d–f; Table S1). However, 18S rRNA transcript and mRNA taxonomy indicated stronger drought impact on eukaryotic compositions especially in the coastal fen (Table S1).

Random forest was employed to compare the accuracy of microbiome changes in predicting dry and wet months between different proxies. The accuracies of the proxies are in the following order: mRNA function ≥ mRNA taxonomy > rRNA transcript > rRNA gene for prokaryotes (Fig. 2a and c); rRNA transcript ≥ mRNA taxonomy > mRNA function and rRNA gene for eukaryotes (Fig. 2b and d). This comparison suggests that mRNA proxies are better in revealing prokaryotic microbiome dynamics while taxonomy-based RNA proxies perform better for eukaryotic microbiomes. The limitation of the current databases for eukaryotic functions may lead to the weaker performance of mRNA function for eukaryotes, but it might also be due to the shared same basic metabolic activities among eukaryotes [13, 14]. The poor performance of DNA proxies in both cases suggests that 16S and 18S rRNA genes that have been widely used may not be sufficient in revealing the microbiome changes in a dynamic environment.

**Fig. 2:**
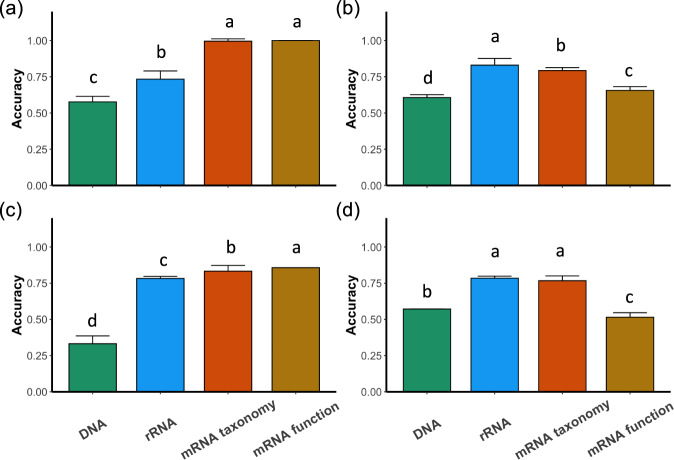
Accuracy of microbiome changes in predicting dry and wet months under different proxies. **a** Accuracy of prokaryotic microbiome changes in predicting in the coastal fen. **b** Accuracy of eukaryotic microbiome changes in predicting in the coastal fen. **c** Accuracy of prokaryotic microbiome changes in predicting in the percolation fen. **d** Accuracy of eukaryotic microbiome changes in predicting in the percolstion fen. The values are shown as mean + standard deviation (*n* = 100). Different letters indicate significant differences between different proxies (Kruskal-Wallis posthoc dunn test adjusted *P* < 0.05). DNA indicates 16S/18S rRNA genes, while rRNA indicates 16S/18S rRNA transcripts.

Remarkably, both prokaryotic and eukaryotic microbiome compositions showed a hydrology-related (wet vs. dry months) change based on corresponding proxies (Fig. 1). These changes were weaker in percolation fen (Table S1), probably due to the weaker decline of the water level in the dry months (Fig. S1). However, compared with the previous year without drought [8], these patterns were much stronger in both sites, illustrating that microbiomes are sensitive to (extreme) weather changes in rewetted fens. To gain insights from the functional level, the mRNA transcripts related with stress responses annotated with SEED [15] were investigated. Most of the stress-response-functions (including those oxygen- and temperature-sensitive ones) showed a significant increase in the dry months (Fig. S2), highlighting a mechanistic response of microbiomes to summer drought.

Our study compared four different proxies for microbiome structure and function, suggesting that the choice of proxy matters in revealing microbiome dynamics. With corresponding proxies, we found that both prokaryotic and eukaryotic microbiomes showed a strong response to summer drought in two rewetted fens. Future microbiome analyses are needed with integrated process data to understand the related ecological consequences.

## Supplementary Information

Supporting Information

## Data Availability

All the sequencing data are available through the European Nucleotide Archive of European Molecular Biology Laboratory with the study accession number PRJEB51908.
