# Supplementary material for: The impact of summer drought on peat soil microbiome structure and function-A multi-proxy-comparison
Source: ISME Commun. 2022 Aug 25;2:78. doi: 10.1038/s43705-022-00164-x (PMC9723574; doi:10.1038/s43705-022-00164-x)
Supplement: Supplementary file 1 — Supporting Information [file 43705_2022_164_MOESM1_ESM.pdf]

Supporting Information

The impact of summer drought on peat soil microbiome structure and function--A multi-proxy-comparison

Haitao Wang[1], Mareike Meister[2], Corinna Jensen[3], Andreas W. Kuss[3], Tim Urich[1]*

1. Institute of Microbiology, University of Greifswald, Greifswald, Germany
2. Diabetes Competence Centre Karlsburg (KDK), Leibniz Institute for Plasma Science and Technology (INP), Karlsburg, Germany
3. Human Molecular Genetics Group, Department of Functional Genomics, University Medicine Greifswald, Greifswald, Germany

*Corresponding to: haitao.wang@uni-greifswald.de or tim.urich@uni-greifswald.de

Materials and methods

Study sites and soil sampling

The two study sites namely coastal fen and percolation fen are located in Mecklenburg-Western Pomerania in Northeastern Germany. A detailed description of these two sites regarding the land-use history, management and water characteristics can be found in [1]. Peat soil samples were taken consistently since April 2017 and the sampling method was described in [2]. Additionally, a subset of soil samples was transferred into RNase-free Eppendorf tubes, which were then transported to the lab with dry ice. These samples were stored in -70 °C until further processing. The current study focuses on topsoil (5-10 cm) samples from April, June, August, October of 2018, and February of 2019, covering the whole period when drought was happening in 2018, which is reflected in the water level and redox potential changes (Fig. S1). In total, 30 samples (2 sites × 5 time points × 3 replicates) were used for amplicon and metatranscriptomic sequencings.

DNA extraction, amplicon sequencing and data processing

The extraction of total DNA as well as 16S rRNA and 18S rRNA gene amplicon sequencings were performed as previously described [2]. All the sequencing data were deposited in the European Nucleotide Archive of European Molecular Biology Laboratory (EMBL) with the study accession number PRJEB51908.

16S rRNA and 18S rRNA gene amplicon sequences were processed according to [2]. Briefly, the raw sequences were demultiplexed, trimmed and processed with *dada2* (v1.8.0) pipeline [3] in R v3.6.3. After removing the chimeras, the representative sequences of amplicon sequencing variants (ASVs) were used as queries for BLASTN searches against a modified version of SILVA 128 database [4]. Taxonomy was assigned to each ASV using lowest common ancestor (LCA) algorithm (Min Score 155; Max Expected 0.01; Top Percent 2.0; Min Support 1) with MEGAN5 [5]. The singleton ASVs were removed. ASVs of the 16S rRNA gene assigned as chloroplast or mitochondria were also removed.

To mitigate the impact of uneven sequencing depths, tables of ASV counts were normalized using metagenomeSeq's CSS [6]. Bray-Curtis dissimilarity distances were calculated based on the normalized tables. Then the Principal Coordinates Analysis (PCoA) was conducted to investigate changes in community compositions. Permutational multivariate analysis of variance (PERMANOVA) was performed to test the significance of the impact of drought (Wet: 18-April

and 19-Feb; Dry: 18-Jun, 18-Aug and 18-Oct) and variations explained on community compositions. The homogeneity of dispersions was tested with *betadisper* followed by analysis of variance (ANOVA), comparing the mean distance-to-centroid among different time points to evaluate the dispersion impact. The statistics were done using the *vegan* package.

RNA extraction, library preparation and metatranscriptomic sequencing

The total RNA was extracted from 0.5-1.0 g soil using the RNeasy® PowerSoil® Total RNA Kit (QIAGEN, Hilden, Germany) according to the manufacturer's protocol with some modification. Vortex in the bead-beating step was replaced with a FastPrep®-24 5G instrument (MP Biomedicals, Santa Ana, USA), with an intensity of 5.5 m/s for 30 s. The extracted RNA was treated with DNase I (Zymo Research, Freiburg, Germany) to remove DNA, followed by cleaning with the MEGAclear™ Kit (Thermo Fisher Scientific, Waltham, MA, USA). The quality of cleaned RNA was checked by 2100 Bioanalyzer (Agilent, Santa Clara, CA, USA) and the concentration of cleaned RNA was determined with Qubit® RNA BR Assay Kit (Invitrogen, Waltham, MA, USA). To enrich the mRNA fraction, 42.5 ng of cleaned RNA were used for amplification with the MessageAmp™ II-Bacteria RNA Amplification Kit (Thermo Fisher Scientific, MA, USA). 100 ng of the amplified RNA (aRNA) were used for the sequencing library preparation with NEBNext® Ultra™ II RNA Library Prep Kit for Illumina® (New England Biolabs, Ipswich, MA, USA). Library preparation was processed according to the manufacturer's protocol with some modifications. The fragmentation time in step 4.1 was changed to 3 min for aRNA. HighPrep™ PCR beads (MagBio Genomics Inc., Gaithersburg, USA) were used to replace the SPRIselect beads and a size selection (aiming size: 250 bp) was introduced. The final libraries were sequenced with an Illumina Next Seq 550 System (paired end; 2 × 150 bp) using one NextSeq 500/550 High Output Kit v2.5 (300 Cycles) and one NextSeq 500/550 Mid Output Kit v2.5 (300 Cycles) (Illumina, San Diego, CA, USA).

The metatranscriptomic sequencing data were submitted to the European Nucleotide Archive of EMBL with the study accession number PRJEB51908.

Metatranscriptomic data processing

The raw forward and reverse sequences were merged with a minimum overlap of 10 bp using FLASH [7]. The poly-A/T tail with a minimum length of 15 bp was trimmed on both ends of the merged sequences, and sequences with quality score mean < 25 were filtered out using PrinseqLite

[8]. SortMeRNA (v2.1) [9] was then used to categorize the sequences into small subunit (SSU) rRNA, large subunit (LSU) rRNA and non-rRNA (putative-mRNA) fractions. SSU rRNA sequences (with a length of 230-250 bp) were then subsampled to 200,000 sequences for each sample using USEARCH [10]. The taxonomy assignment was performed with modified SILVA 128 database using LCA algorithm as described above. Reads of the putative-mRNA fraction were aligned to protein sequences against the NC_nr database (accessed 12/04/2020) using DIAMOND [11]. Taxonomies and functions were assigned to reads with hits using LCA algorithm (Min Score 155; Max Expected 0.01; Top Percent 4.0; Min Support 1) with MEGAN6 [12]. The function profile was assigned with SEED database [13].

One sample (from percolation fen October) was removed from the downstream analysis as this sample showed a huge distinction from the other two replicates, which might be due to a contamination during sample processing. The community compositions of prokaryotes and eukaryotes on RNA level were analyzed using the same method as mentioned above, based on SSU rRNA (taxonomy), mRNA taxonomy and mRNA function. The statistics were also performed accordingly as described above. The dispersions of coastal fen samples (prokaryotes) and percolation fen samples (eukaryotes) based on mRNA function are inhomogeneous. However, the PCoA plots indicate that the dispersion effect is much smaller than the drought effect (Fig. 1f). In addition, the proxies were quantitatively evaluated with Random Forest by comparing the accuracy of microbiome changes in predicting the drought events using *randomForest*, *caret* and *rfUtilities* packages. To get rid of the randomness impact, 100 random forests were run for each proxy, and accuracies of the proxies were statistically compared with Kruskal-Wallis posthoc dunn tests using *PMCMR* package. Further, some metabolic functions related with stress responses as identified by SEED database and their changes in absolute abundances were shown, and the significance of the difference between dry and wet months were evaluated with Kruskal-Wallis test using *vegan* package. The absolute abundance was calculated using total RNA content according to Söllinger et al. 2018 [14].

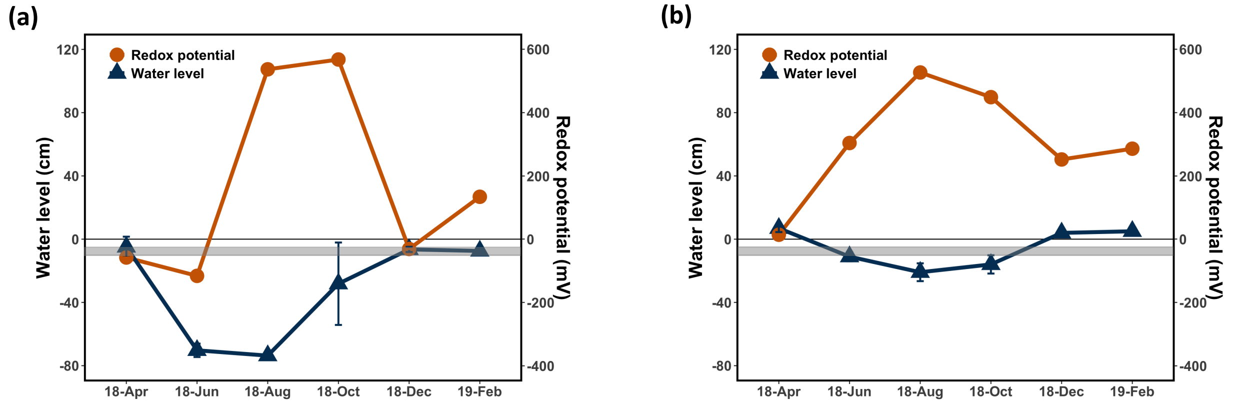

Fig. S1 Changes in water level and redox potential over times in coastal fen (a) and percolation fen (b). The water level was scanned every minute and recorded at 15-min intervals on a Campbell Scientific CR300 datalogger (Logan, USA). The redox potential was measured every time when soil samples were taken with ecoTech redox electrodes (Bonn, Germany) and the data was recorded by a Greisinger GMH3531 reader (Regenstauf, Germany) at the depth of 10 cm. Water level is shown as the monthly mean ± standard deviation, while redox potential was measured when the soil samples were taken in different months. The horizontal grey bar indicates the sampling depth (5-10 cm). Kruskal-Wallis test showed that the water level in both sites was significantly different between months ($P<0.001$).

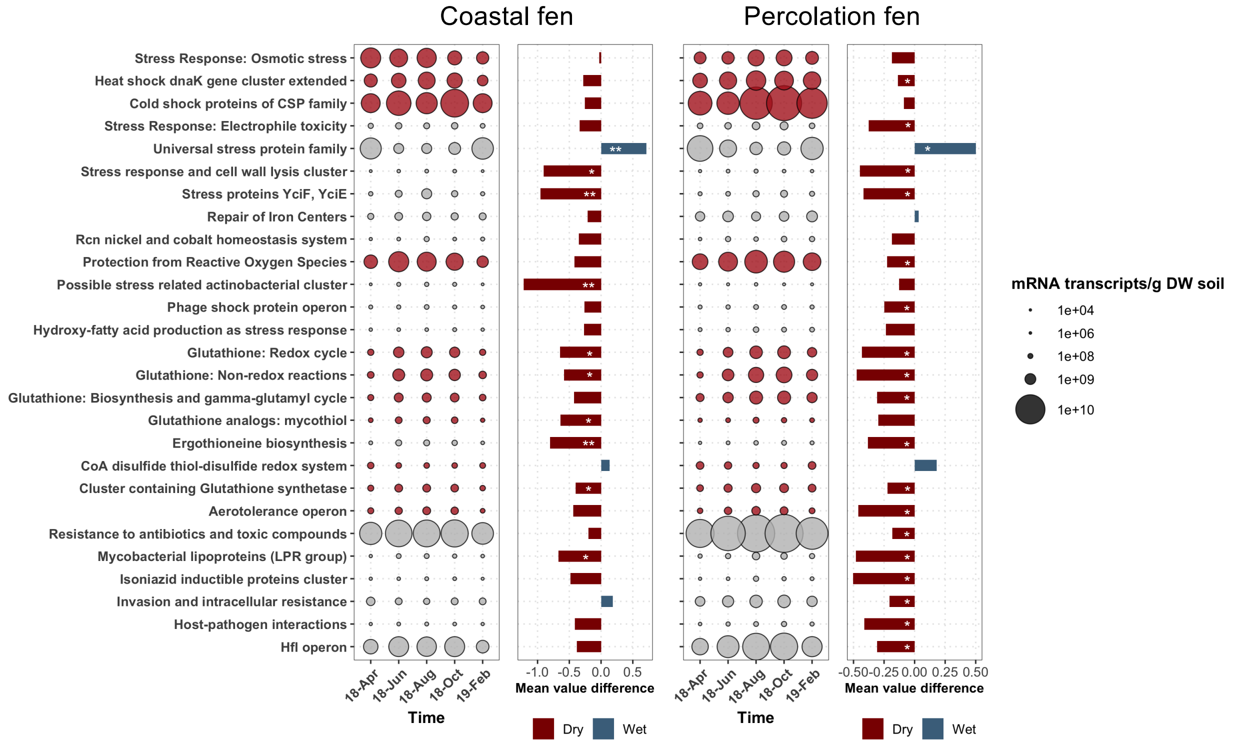

Fig. S2 Bubble plots show changes in the abundance of functions (mRNA transcripts g^{-1} dry soil) related with stress response characterized by SEED database. The red colored bubbles indicate oxygen- and temperature-sensitive functions. Bar plots show the mean value changes of the abundance between dry and wet months, calculated as $\log_{10}$(abundance in wet months)-$\log_{10}$(abundance in dry months). Therefore, negative values mean higher abundance in dry months while positive values mean higher abundance in wet months. The asterisks indicate the significant level as characterized by Kruskal-Wallis test (*P<0.05, **P<0.01, P values were adjusted with false discovery rate method). DW, dry weight.

Table S1 PERMANOVA showing the effect of drought on microbiome compositions using different proxies

		**Prokaryotes**		**Eukaryotes**	
		**R^2**	***P***	**R^2**	***P***
C	rRNA gene (DNA)	0.05	0.758	0.12	0.009**
	SSU rRNA	0.11	0.040*	0.15	0.001***
	mRNA taxonomy	0.39	0.001***	0.19	0.020*
	mRNA function	0.46	0.001***	0.11	0.007**
P	rRNA gene (DNA)	0.06	0.768	0.09	0.148
	SSU rRNA	0.09	0.199	0.12	0.049*
	mRNA taxonomy	0.21	0.021*	0.14	0.076
	mRNA function	0.29	0.006**	0.11	0.011*

Significance level, 0.001***, 0.01**, 0.05*; C, coastal fen; P, percolation fen.

References

1. Jurasinski G, Ahmad S, Anadon-Rosell A, Berendt J, Beyer F, Bill R, et al. From Understanding to Sustainable Use of Peatlands: The WETSCAPES Approach. Soil Systems. 2020;4:14.
2. Wang H, Weil M, Dumack K, Zak D, Munch D, Gunther A, et al. Eukaryotic rather than prokaryotic microbiomes change over seasons in rewetted fen peatlands. FEMS Microbiol Ecol. 2021;97:fiab121.
3. Callahan BJ, McMurdie PJ, Rosen MJ, Han AW, Johnson AJA, Holmes SP. DADA2: high-resolution sample inference from Illumina amplicon data. Nat Methods. 2016;13:581-83.
4. Lanzén A, Jørgensen SL, Huson DH, Gorfer M, Grindhaug SH, Jonassen I, et al. CREST–classification resources for environmental sequence tags. PloS One. 2012;7:e49334.

5. Huson DH, Mitra S, Ruscheweyh H-J, Weber N, Schuster SC. Integrative analysis of environmental sequences using MEGAN4. Genome Res. 2011;21:1552-60.
6. Paulson JN, Stine OC, Bravo HC, Pop M. Differential abundance analysis for microbial marker-gene surveys. Nat Methods. 2013;10:59-60.
7. Magoč T, Salzberg SL. FLASH: fast length adjustment of short reads to improve genome assemblies. Bioinformatics. 2011;27:2957-63.
8. Schmieder R, Edwards R. Quality control and preprocessing of metagenomic datasets. Bioinformatics. 2011;27:863-64.
9. Kopylova E, Noé L, Touzet H. SortMeRNA: fast and accurate filtering of ribosomal RNAs in metatranscriptomic data. Bioinformatics. 2012;28:3211-17.
10. Edgar RC. Search and clustering orders of magnitude faster than BLAST. Bioinformatics. 2010;26:2460-61.
11. Buchfink B, Xie C, Huson DH. Fast and sensitive protein alignment using DIAMOND. Nat Methods. 2015;12:59-60.
12. Huson DH, Beier S, Flade I, Górska A, El-Hadidi M, Mitra S, et al. MEGAN community edition-interactive exploration and analysis of large-scale microbiome sequencing data. PLoS Comput Biol. 2016;12:e1004957.
13. Overbeek R, Begley T, Butler RM, Choudhuri JV, Chuang H-Y, Cohoon M, et al. The subsystems approach to genome annotation and its use in the project to annotate 1000 genomes. Nucleic Acids Res. 2005;33:5691-702.
14. Söllinger A, Tveit AT, Poulsen M, Noel SJ, Bengtsson M, Bernhardt J, et al. Holistic assessment of rumen microbiome dynamics through quantitative metatranscriptomics reveals multifunctional redundancy during key steps of anaerobic feed degradation. mSystems. 2018;3:e00038-18.
